# *Plasmodium vivax* severe imported malaria in two migrants in France

**DOI:** 10.1186/s12936-019-3067-5

**Published:** 2019-12-16

**Authors:** Arezki Izri, Sandrine Cojean, Claire Leblanc, Yves Cohen, Olivier Bouchaud, Rémy Durand

**Affiliations:** 1 Service de Parasitologie- Mycologie, CHU Avicenne, Assistance Publique-Hôpitaux de Paris, 125 rue de Stalingrad, 93009 Bobigny Cedex, France; 2 Unité des Virus Émergents (UVE: Aix-Marseille Univ-IRD 190-Inserm 1207-IHU Méditerranée Infection), Marseille, France; 3 UFR SMBH, Université Paris 13, Bobigny, France; 4 UMR 8076 CNRS BioCIS, Université Paris-Sud, Université Paris-Saclay, Châtenay-Malabry, France; 5 Centre National de Référence du Paludisme, hôpital Bichat-Claude Bernard, APHP, Paris, France; 6 Service de Pédiatrie générale, CHU Jean Verdier, Bondy, France; 7 Réanimation Médico-Chirurgicale, CHU Avicenne, Bobigny, France; 8 Service de Maladies Infectieuses et Tropicales, CHU Avicenne, Bobigny, France

**Keywords:** *Plasmodium vivax*, Imported malaria, Severe malaria, Relapses

## Abstract

**Background:**

With less than one severe case per year in average, *Plasmodium vivax* is very rarely associated with severe imported malaria in France. Two cases of *P. vivax* severe malaria occurred in patients with no evident co-morbidity. Interestingly, both cases did not occur at the primary infection but during relapses.

**Case presentations:**

Patient 1: A 27-year old male, born in Afghanistan and living in France since 2012, was admitted on August 2015 to the Avicenne hospital because of abdominal pain, intense headache, fever and hypotension. The patient was haemodynamically unstable despite 5 L of filling solution. A thin blood film showed *P. vivax* trophozoites within the red blood cells. To take care of the septic shock, the patient was given rapid fluid resuscitation, norepinephrine (0.5 mg/h), and intravenous artesunate. Nested polymerase chain reactions of the SSUrRNA gene were negative for *Plasmodium falciparum* but positive for *P. vivax.* The patient became apyretic in less than 24H and the parasitaemia was negative at the same time. Patient 2: A 24-year old male, born in Pakistan and living in France, was admitted on August 2016 because of fever, abdominal pain, headache, myalgia, and nausea. The last travel of the patient in a malaria endemic area occurred in 2013. A thin blood film showed *P. vivax* trophozoites within the red blood cells. The patient was treated orally by dihydroartemisinin-piperaquine and recovered rapidly. Nine months later, the patient returned to the hospital with a relapse of *P. vivax* malaria. The malaria episode was uncomplicated and the patient recovered rapidly. Three months later, the patient came back again with a third episode of *P. vivax* malaria. Following a rapid haemodynamic deterioration, the patient was transferred to the intensive care unit of the hospital. In all the patient received 10 L of filling solution to manage the septic shock. After 5 days of hospitalization and a specific treatment, the patient was discharged in good clinical conditions.

**Conclusion:**

Clinicians should be aware of the potential severe complications associated with *P. vivax* in imported malaria, even though the primary infection is uncomplicated.

## Background

France is the European country reporting the highest number of imported malaria cases, with an estimated number of cases exceeding 4000 each year [1]. Between 2005 and 2015, the countries with the highest average number of reported cases per year were France (2169 cases), United Kingdom (1898 cases) and Italy (637 cases). For comparison, USA reported 1511 cases [2]. In France, more than 85% of cases are caused by *Plasmodium falciparum*, followed by *Plasmodium ovale* (5.6%), *Plasmodium vivax* (4%); *Plasmodium malariae* and mixed infections amounting to 1.7% each.

In endemic areas, *P. falciparum* is responsible for the large bulk of the morbidity and mortality of malaria [3] even though it has been shown recently that the morbidity and mortality of *P. vivax* have been underestimated, particularly in patients who have other comorbidities, such as malnutrition, HIV, or coexisting infections [4–7].

In France, *P. falciparum* appears as the species responsible for almost all severe cases and deaths in travelers [1]. With less than one severe case per year in average, *P. vivax* is very rarely associated with severe imported malaria in France. Two cases of *P. vivax* severe malaria occurred in patients with no evident co-morbidity. Interestingly, both cases did not occur at the primary infection but during relapses.

## Case presentations

### Patient 1

A 27-year old male, born in Afghanistan, living in France since 2012 and having not traveled in an endemic area since that date, was admitted on 2nd of August 2015, 24 h after the symptoms onset, to the emergency unit of the Avicenne hospital because of abdominal pain, intense headache, fever and fatigue. At presentation, he was febrile (38.9 °C), hypotensive (79/48 mmHg) and tachycardic (110 beats per minute). There were no meningeal signs and the Glasgow Coma Score was normal (15/15). The patient was hemodynamically unstable despite 5 L of filling solution. Oxygen saturation while breathing ambient air was 94% (PO_2_ 80 mmHg). The lactatemia was 2.5 mmol/L and total bilirubinaemia was 20 μmol/L. Abdominal–thorax–pelvis computed tomography was done in emergency and was normal. He was then transferred to the intensive care unit (ICU) of the hospital. Severe malaria was suspected and a thin blood film fixed with pure methanol and stained with Diff-Quick showed *P. vivax* trophozoites within the red blood cells (parasitaemia 0.3%). Laboratory examination revealed disseminated intravascular coagulation with a platelet count of 37 × 10^9^/L, a diminished PT (53%) and fibrinogen (1.65 g/L) and a high d-Dimer concentration (6595 ng/mL). A moderate hepatic cytolysis (aspartate aminotransferase, 76 IU/L and alanine aminotransferase, 148 IU/L), and a systemic inflammation (C-reactive protein, 19 mg/L and procalcitonine, 9.33 µg/L) were also shown. To take care of the septic shock, the patient was given rapid fluid resuscitation, norepinephrine (0.5 mg/h), intravenous artesunate, cefotaxime, metronidazole and gentamicine. No other infections were identified despite microbiological investigations including blood and urine cultures. Serological tests for HIV1&2 were negative. He did not smoke or drink alcohol and he declared not to use other drugs. No comorbidities were known for this subject otherwise healthy. Nested polymerase chain reactions (PCRs) of the SSUrRNA gene with specific species primers [8] were performed at the French Malaria Reference Center and were negative for both *P. falciparum* and *Plasmodium knowlesi,* but positive for *P. vivax.* The patient became apyretic in less than 24H and the parasitaemia was negative at the same time. He was then transferred to the Department of Infectious and Tropical Diseases where he was given chloroquine orally (25 mg/kg on 3 days). Antibiotics were stopped on day 3. After 5 days of hospitalization, the patient was discharged in good clinical conditions. A follow up performed 2 days later showed no parasites on thin and thick blood films and the patient remained apyretic. The measured G6PD enzyme level of the patient being normal, a radical treatment with primaquine was proposed to the patient who declined the proposition. Two other relapses occurred 10 weeks and 16 weeks later but without criteria of severity. A radical treatment with primaquine, 30 mg per day for 2 weeks, was again proposed to the patient who followed it successfully.

### Patient 2

A 24-year old male, born in Pakistan and living in France since 2010, was admitted on 15th of August 2016 to the emergency unit of our hospital for fever, abdominal pain, headache, myalgia, and nausea. The last travel of the patient in a malaria endemic area (Pakistan) occurred in 2013. Laboratory examination revealed a thrombopenia (platelet count, 61 × 10^9^/L), a systemic inflammation (C-reactive protein, 145 mg/L), and a thin blood film showed *P. vivax* trophozoites within the red blood cells (parasitaemia 0.22%). The patient was hospitalized due to abnormalities in biological parameters including hyperbilirubinaemia (102 μmol/L). The patient received intravenous quinine on Day 0, due to uncontrollable vomiting, and, according to the French recommendations, was treated orally by artenimol–piperaquine the three following days [9]. Then the patient recovered rapidly. The measured G6PD enzyme level of the patient being normal, a radical treatment with primaquine was proposed to the patient who declined the proposition.

The patient returned to the hospital more than 9 months later (on 29th of May 2017) when he presented with a relapse of *P. vivax* malaria (parasitaemia 0.3%). The patient had still not travelled in a malaria endemic area since his last hospitalization. The malaria episode was uncomplicated. As the patient presented again with vomiting he was given intravenous quinine on Day 0 and a total dose of 25 mg/kg chloroquine administered over 3 days.

Three months later, 48 h after the symptoms onset, the patient came back again for fever (38.5 °C), headache, arthralgia, myalgia, abdominal pain and subsequently, vomiting and diarrhoea. A thin blood film revealed the presence of *P. vivax* trophozoites (parasitaemia 0.38%). Following a rapid haemodynamic deterioration, the patient was transferred to the ICU. At the admission, the patient was hypotensive (92/48 mmHg) and tachycardic (100 beats per minute). The Glasgow Coma Score was normal (15/15). Lactataemia was 2.3 mmol/L and total bilirubinemia was 52 μmol/L. In all the patient received 10 L of filling solution to manage the septic shock. As the patient suffered from uncontrollable vomiting he was given intravenous quinine on Day 0 and was treated orally by chloroquine the three following days. No other infections were identified despite extensive microbiologic investigations. However, the patient received cefotaxime and gentamycin on Day 0 which were stopped on day 3. Nested polymerase chain reactions (PCRs) of the SSUrRNA gene with specific species primers were negative for both *P. falciparum* and *P. knowlesi,* but positive for *P. vivax.* After 5 days of hospitalization, the patient was discharged in good clinical conditions. A follow up performed 2 days later showed no parasites on thin and thick blood films and the patient remained apyretic. After two other relapses, which were uncomplicated, occurring in the following months, a radical treatment with primaquine, 30 mg per day for 2 weeks, was once again proposed to the patient who was then compliant.

## Discussion and conclusions

*Plasmodium vivax* is increasingly recognized as responsible for severe malaria in endemic areas and also in imported malaria [10, 11]. In a recent retrospective study conducted in Sweden, Wangdahl et al. claimed that 7.7% of imported *P. vivax* cases were severe, which was comparable to the proportion seen with *P. falciparum* (9.4%) [11]. Those surprising results may reflect an increase or a better diagnosis of severe cases due to *P. vivax* during the last decades. However, as single nor mixed infections in their series were not systematically confirmed by PCR, it is possible that the number of severe *P. vivax* cases was over estimated due to unrecognized association with *P. falciparum*. In the both cases presented here, it was confirmed by PCR assays that *P. vivax* was the unique species involved in the malaria episodes.

Both patients suffered from septic shock, which is a criterion frequently reported for *P. vivax* severe malaria [7, 11, 12]. Patient 2 showed a marked hyperbilirubinaemia (102 μmol/L) on his first visit as a clinically uncomplicated malaria episode was diagnosed. The current World Health Organization criteria for severe forms of malaria include hyperbilirubinaemia with a threshold > 50 μmol/L [13, 14]. We agree with other authors that hyperbilirubinaemia, when isolated, does not seem to be a suitable criterion for imported severe malaria [11]. At his third visit, as patient 2 developed a severe episode, the bilirubinaemia was slightly above the threshold (52 μmol/L). Patient 1 had a value below the threshold (20 μmol/L) during his severe episode.

In nonendemic areas, older age is a risk factor for severe malaria for *P. falciparum* and for other species [11, 15]. It may be underlined that both patients were young and thus were not particularly at risk for this point.

According to the current literature, imported malaria episodes due to other species than *P. falciparum* usually do not evolve towards the death of travellers [11, 16], but in some instances they may have led to serious complications and important residual damages [17]. As their condition deteriorated, both patients presented here were rapidly admitted in ICU where they received appropriate treatments. Thus, both patients recovered without sequelae.

Both patients had not travelled recently in a malaria endemic area and did not live near an airport. None of the patients had reported an episode of severe malaria when they were still in endemic area. *Plasmodium vivax* is prevalent in Afghanistan and Pakistan and hepatic dormant forms (hypnozoites) may persist several years in the liver of infected subjects [18, 19]. There are different strains of *P. vivax* according to geographical region/endemicity areas, with relapse patterns that vary by latency (time to first relapse), likelihood of relapse, and frequency of relapses [19]. The temperate strains, including those found in Afghanistan and in the North of Pakistan, relapse much more slowly than other strains (up to 2 years or more) [19]. They may also exhibit extended incubation period (7–14 months) [18, 20, 21]. Much longer incubations (> 3 years) are exceptionally reported for *P. vivax* in the literature [22]. It was inferred that Patient 1 presented at his first visit to the Avicenne hospital during a relapse because he had not travelled in endemic area since at least 3 years. However, as the primary episode was not observed at the hospital, an exceptionally long incubation cannot be formally excluded for this case.

The cases presented here are particular in that the severity of the attacks revealed itself not at the primary infection but on relapses. Severe *P. vivax* episodes have been already reported in endemic areas after 2–3 relapses in the case of strains with long incubation [22]. According to the current literature, such cases have not been reported previously in imported malaria. It is generally admitted that a relapse originates from a single genotype which is dormant in the liver [23, 24]. As infections are often polyclonal [25–27], the clonal parasite population responsible of the relapse may be different from the one giving the primary infection or other subsequent relapses. Relapses may also result from activation of heterologous latent hypnozoites acquired from previous inoculations [28]. Thus, the parasites responsible for a severe relapse may be more virulent than the parasites observed during other episodes. It is also possible that the patients experienced a degradation of their health during their stay in France due to difficult living conditions of immigrants or refugees.

The risks of relapses were explained to both patients at their first stay in hospital but they accepted to take the radical cure only after several malaria episodes. In this context, a more persuasive explanation, taking into account the language barrier, could have been beneficial to patients.

In conclusion, clinicians should be aware of the potential severe complications associated with *P. vivax* in imported malaria, even though the primary infection is uncomplicated. A radical treatment with primaquine or tafenoquine should be implemented whenever possible in order to avoid relapses, severe or not [29, 30].

## Data Availability

Not applicable.

## Abbreviations

ICU: intensive care unit
PCR: polymerase chain reaction
PT: prothrombin time
SSUrRNA: small subunit ribosomal ribonucleic acid
WHO: World Health Organization
